# Exploring the impact of mental health conditions on vaccine uptake in high-income countries: a systematic review

**DOI:** 10.1186/s12888-022-04512-y

**Published:** 2023-01-07

**Authors:** Anne M. Suffel, Oyinkansola Ojo-Aromokudu, Helena Carreira, Sandra Mounier-Jack, David Osborn, Charlotte Warren-Gash, Helen I. McDonald

**Affiliations:** 1 Department of Infectious Disease Epidemiology, London School of Hygiene and Tropical Medicine, London, UK; 2 Department of Global Health and Development, London School of Hygiene and Tropical Medicine, London, UK; 3 Department of Non-Communicable Disease Epidemiology, London School of Hygiene and Tropical Medicine, London, UK; 4 Division of Psychiatry, University College London, London, UK

**Keywords:** Mental health, Vaccination, Health inequality, High-income countries

## Abstract

**Background:**

Vaccination is an essential public health intervention to reduce morbidity and mortality from infectious diseases. Despite being at higher at risk of infectious diseases, health inequalities towards vaccine uptake in people with mental health issues have not been systematically appraised.

**Methods:**

We searched 7 databases from 1994 to 26/03/2021. We included all studies with a relative measure of effect comparing a group with a mental health issue to a control group. All studies covering any mental health issue were eligible with no constraints to study population, vaccine type or region, provided in a high-income country for comparability of health care systems. The study outcomes were synthesised by study population, mental health issue and type of vaccine.

**Results:**

From 4,069 titles, 23 eligible studies from 12 different countries were identified, focusing on adults (*n* = 13) or children (*n* = 4) with mental health issues, siblings of children with mental health issues (*n* = 2), and mothers with mental health issue and vaccine uptake in their children (*n* = 6). Most studies focused on depression (*n* = 12), autism, anxiety, or alcoholism (*n* = 4 respectively). Many studies were at high risk of selection bias.

**Discussion:**

Mental health issues were associated with considerably lower vaccine uptake in some contexts such as substance use disorder, but findings were heterogeneous overall and by age, mental health issue or types of vaccine. Only individuals with mental health issues and physical comorbidities had consistently higher uptake in comparison to other adults.

Mental health should be considered as a health inequality for vaccine uptake but more context specific research is needed focusing more on specific mental health issues and subgroups of the population to understand who misses vaccination and why.

**Supplementary Information:**

The online version contains supplementary material available at 10.1186/s12888-022-04512-y.

## Introduction

Mental health issues do not only lower well-being and quality of life directly [1] but also affect physical health and life expectancy [2, 3]. These health inequalities apply to many people as mental health issues are very common: A global meta-analysis by Steel et al. [4] showed that 17.6% of the global population have met diagnostic criteria of a common mental disorder within the last 12 months and around 30% of the population have experienced a mental disorder at least once in their lives.

This disparity of physical health between people with mental health issues and without might be partly due to inequalities in healthcare access and utilization. People with severe mental illness (major depressive disorder, schizophrenia, bipolar disorder) suffer from greater health disparity due to inequalities in health care access and utilization [5], and people with common mental disorders experience problems with accessing primary care services [6].

Although many studies focused on non-communicable diseases and unhealthy lifestyle in people with mental health issues [5], these individuals also have a higher risk for infectious diseases which is probably driven by a combination of many environmental and social risk factors, but also a potentially higher genetic susceptibility [7]. This makes vaccine uptake a crucial public health intervention for people with mental health problems.

Previous reviews have not investigated health inequalities concerning mental health issues and vaccine uptake in a systematic way [8] or have focused on the uptake of influenza or pneumococcal vaccine only [9, 10]. Consequently, this systematic review is the first study systematically exploring the impact of mental health issues on the choice and access to vaccination services– covering several types of vaccination, different types and definitions of mental health issues, and across different subgroup of the population, such adults at different ages in community or care home settings, or parents making decisions about the vaccination for their children. We focused on high-income settings where routine-access to vaccination services is usually available.

## Methods

Our systematic review followed the Preferred Reporting Items for Systematic Reviews and Meta-Analyses (PRISMA) guidelines [11]. A review protocol was developed a priori and registered with the International prospective register of systematic reviews (PROSPERO) on the 26^th^ of March 2021 (registration number CRD42021245322). The full protocol can be found in the supplementary material.

### Population

The studies of interest in this systematic review were those that included any type of population who has access to a routinely delivered vaccination programme in a high-income country, as defined by the World Bank [12], of any age, or population subgroup. This also includes groups such as refugees and homeless people in cases with a vaccination programme for those groups in place.

### Exposure of interest

We included studies covering a broad range of mental health issues, such as diagnoses covered in the 10^th^ edition of the International Classification of Diseases (ICD-10) (e.g., major depressive disorder, autism spectrum disorder, etc.) or symptom-based reports (e.g., items from the SF-12 [13] or PROMIS Global-10 [14], etc.) and severe psychological distress.

Although we included studies with alcohol dependence or abuse defined as such by the study authors, we did not include studies reporting smoking and drinking behaviours per se because definitions of abnormal substance use are highly influenced by context and social norms. Furthermore, we did not include studies covering potential risk factors for mental health issues such as family stress and including life-circumstances such as prison detention which as usually closely linked to mental health issues.

For our analysis, we grouped the mental health issues into depressive disorders, anxiety (including mixed anxiety and depression), substance use disorder and alcohol abuse, autism spectrum disorders and severe mental illness including bipolar disorder, schizophrenia and other forms of psychoses.

### Control group

We included a control without mental health issue or the general population. In case–control studies, a vaccinated versus a non-vaccinated group should be compared with mental health as a covariate in the statistical model.

### Outcomes of interest

We included all studies covering the uptake and timing of a recommended vaccine for an individual or a relative with a mental health issue in comparison to a control group.

### Study eligibility

Every type of observational study published in a peer-reviewed journal was eligible for inclusion, e.g., cohort studies, cross-sectional studies, case–control studies. Conference abstracts and PhD theses were also eligible if they contained enough information which was the only deviation from the PROSPERO protocol. We made this decision during the search and screening process as several of these studies added value to the broad range of study populations and mental health conditions and seemed of comparable study quality to other studies published in journals.

Any type of relative measure of effect or enough information to calculate a relative measure of effect was sufficient for inclusion. There were no constraints to language of the studies.

### Information sources and search strategy

We searched Embase, MEDLINE and PsycINFO via Ovid, CINAHL via EBESCO, the Cochrane Library, Scopus and Open grey for studies published from 1994 up to the 26/03/2021. The study inclusion was limited to 1994 and later, as the publication of the Diagnostic and Statistical Manual of Mental Disorders IV (DSM-IV) in 1994 led to a significant change in how mental disorders were defined and diagnosed [15].

The search terms were based on terms covering all mental health issues in the 10^th^ edition of the International Classification of Diseases (ICD-10) and other systematic reviews of mental health issues [10, 16]. The search terms for vaccine uptake were based on search terms used in Jain et al. [17] expanded to cover all vaccines given routinely in high income countries. Terms for preventive health and childcare visits were also included to detect vaccination as part of a preventive care appointment.

The search terms for every database can be found in the supplementary material. Backwards citation tracking was conducted for all included papers and encountered systematic reviews on the topic.

### Data acquisition and extraction

Duplicates were removed using a R-script written by AS (https://github.com/Eyedeet/Systematic_review/tree/main) which uses the revtools R-package [18]. The script removes duplicates in a first step using string matching of the digital object identifiers. In a second step, the titles of the papers are compared using a string distance algorithm and a fuzzy string-matching algorithm. AS tested her deduplication algorithm including different sensitivity parameters of the string distance algorithm and the fuzzy string algorithm against a conventional deduplication algorithm as used in the free software Mendeley.

Every study’s title and abstract were screened by two authors independently (OOA & AS). In case of a disagreement, the study was included into a full-text screening which was conducted by the same two reviewers blinded to each other’s decision. Any difference was resolved through discussion by OOA and AS. The data extraction was conducted by AS using a standardized table. A list of extracted items can be found in the supplementary material. If a study had more than one estimate, we extracted the estimate adjusted for the largest number of confounders and the crude estimate. If the estimates were for different types of mental health issues or different types of vaccination, all estimates were extracted.

The studies were grouped by age (adults over 65, general adult population, mothers and their children, and children), by different mental health issue and by type of vaccine.

### Risk of bias assessment

A tool for assessing the risk of bias was developed based on the ROBINS-I [19] tool and was adapted according to the topic of the review. Detailed decision criteria for each type of bias can be found in the supplement (see Table S.1). The risk of bias assessment was conducted by AS and HM in parallel and blinded to each other’s rating for five studies. As there were no disagreement for the piloted studies, AS continued with the rating of the remaining studies.

Additionally, a funnel plot was used to graphically explore potential including reporting bias [20, 21].

### Data analysis

Crude effect sizes in form of odds ratios and confidence intervals were calculated for all studies which did not provide any adjusted measure of effect based on their proportions of vaccinated individuals in the exposed and control groups. For this purpose, the sample sizes of Howard et al. [22] and Lawrence et al. [23] had to be approximated as it was unclear whether the grouping into mental health issues was mutually exclusive. Studies could present effect estimates for different vaccines and different mental health issues. In order to make different studies better comparable, we averaged the uptake over 3 years for each type of vaccine and mental health condition for the study by Browne et al. [24]. For the Howard et al. [22] paper, we derived the confidence intervals from the given *p*-values.

All results were analysed and presented by the underlying study population grouped by age group (e.g., older adults, adults, children, and mother and children), the underlying mental health issue and type of vaccine. We also explored potentially different outcomes for different definitions of being vaccinated, e.g., vaccinated in the last year or ever vaccinated. If a study had more than one effect estimate for different vaccines or mental health conditions, all of them were presented separately. In studies adjusting for different confounders, we presented the effect estimate adjusting for the maximum number of confounders.

Heterogeneity, clinical and statistical, was deemed too high to reasonably apply meta-analytic methods. A grade approach was used to present the certainty of the evidence.

## Results

### Study characteristics

**Fig. 1:**
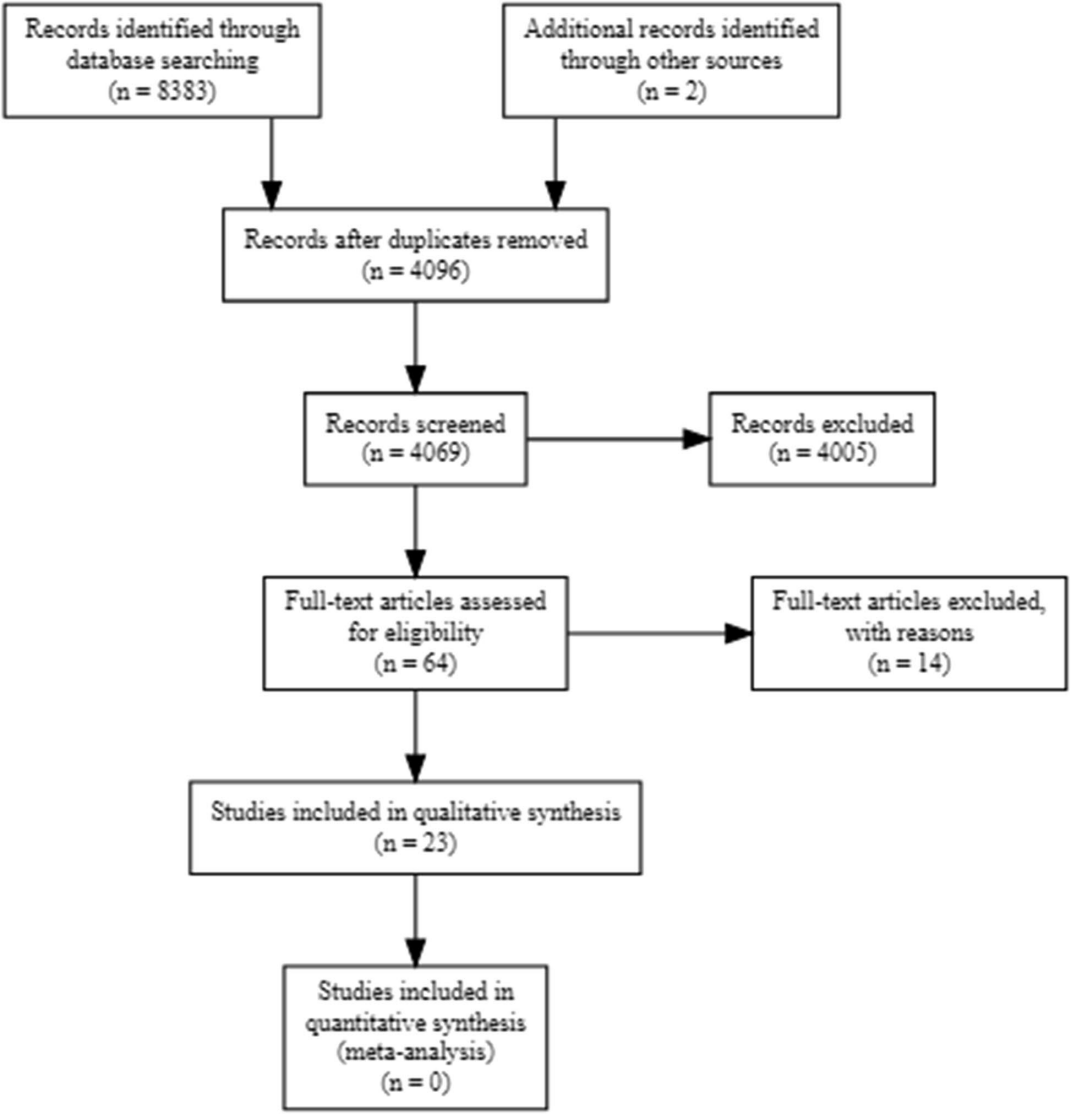
Results from the literature search and screening

A total of 4,069 titles were identified of which 64 were included in the full-text screening resulting in 23 eligible studies published between 2000 and 2021 (see Fig. 1). One of the 23 included studies [25] was identified through backwards citation tracking.

About half of the eligible studies (*n* = 12) were cross-sectional, and 11 of the studies were cohort studies. Studies covered a range of population groups with 12 studies looking at adults over 50 and older, one at the general adult population, four studies at children with mental health issues and six studies exploring the impact of mental health issues in mothers on vaccine uptake in their children. There were 14 studies conducted in the United States, four in the United Kingdom, one single multi-national study in 10 different European countries, and one study each in Australia, Canada, and Denmark. Many studies included more than one mental health issue as exposure and allowed their subjects to fall under more than one category. Depression was the most common mental health issue explored (*n* = 12), followed by autism spectrum disorder (*n* = 4), anxiety (*n* = 4) and substance use disorder including alcoholism (*n* = 4), two studies using general mental health scores, two studies on psychological distress, two studies on dementia, and one study each on eating disorders, psychosis, learning disability, post-traumatic stress disorder. The studies focused on vaccines either given at older age or during childhood: 12 studies looked at influenza vaccine uptake, 4 at pneumococcal vaccine, 10 at different childhood immunizations and one study at tetanus vaccine. The definition and time period of being vaccinated varied between studies as national vaccination schedules differed.

An overview of all included studies and more details about the study population can be found in the in Table 1.

**Table 1:** Study overview

First author, publication year	Country	Design	Definition of exposure	Definition of vaccine receipt	Participants	Overall Population size (N), follow-up time	Gender (% of females) and age breakdown	Statistical analysis method used	Covariates in adjusted model
Elderly									
Bazargan, 2020	United States	Cross-sectional	Depressive symptoms (Geriatric depression scale)	Influenza (ever received)	Older African-Americans living in Los Angeles service area 6 (community recruitment) over 65	Total *N* = 620	65% females, mean age 74 yrs (SD 7.1)	Logistic regression	Age, educational attainment (years of school attendance), living arrangements (alone or partner/family), score for financial difficulty, continuity of medical care, satisfaction with medical care, disability status, self-rated health status, COPD
Browne, 2019	United States	Cohort study	Depression, PTSD, anxiety disorder, serious mental illness (bipolar disorder or schizophrenia), substance use disorder, any mental illness based on ICD-9 codes in outpatient visits	Influenza (last year)	US veterans within the Veterans Health Administration over 50	Total *N* = 4,461,247, Follow-up time: 1 yr	8.5/5.1% females (exp/contr), mean age 55.8/64.7 yrs (SD 13.8/15.1) for exp/contr	Descriptive analysis	None
Buchwald, 2000	United States	Retrospective medical record review	Depression (currently depressed, no further information)	Influenza (ever, last year), PCV (ever)	Natives Americans or Alaska Natives registered with Seattle Indian Health Board over 50	Total *N* = 550	61% females, mean age 60.6 yrs	Logistic regression	Age, sex, marital status, insurance status, alcohol use, other substance use, smoking, purified protein derivative placement, number of health problems, cardiovascular disease, pulmonary disease, diabetes, medication taken
Druss, 2001	United States	Cross-sectional	Psychiatric disorder (excl. substance disorder), substance use disorder, psychiatric disorder (incl. substance use disorder) based on ICD-9 codes	Influenza (ever, last year)	US-veterans registered with the Veterans Health Administration with high-comorbidities	Total *N* = 113,505	21/13.2% females (exp/contr) , mean age 60.8/66 years (SD 12.9/11.5) for exp/contr	Logistic regression	Age, gender, ethnicity, distance to VHA centre, disability caused by military service, health status, facility-level variables, medical comorbidity
Howard, 2006	United States	Cross-Sectional	Depression (self-reported)	Influenza (ever), PCV (ever)	Enrolled patients in Prudential Center for Health care research (US)	Total N = 3,260	56%/58% females, 65/62% 65–75 yrs, 30/31% 75-84yrs, 5/7% > 85yrs (with and without high school education)	Logistic regression	Health literacy, HS degree, age, gender, race, site, comorbidity, income, smoker
Lawrence, 2020	United States	Cross-sectional	Depression, anxiety, substance and drug abuse (results not reported)	Influenza (ever)	Primary care data registry, patients between 60 and 80 years old	Total *N* = 4,120	59.1/44.8% females (exp/contr), mean age 70.8/73.9 yrs for exp/contr	Logistic regression	Socioeconomic status, comorbidity index, diagnosis of influenza, pneumonia or other respiratory diseases, prescription of antidepressants and benzodiazepines, previous vaccine and health care utilisation
Lin, 2016	United States	Cross-sectional	Psychological distress (questionnaire)	Influenza (ever)	Participants from the medical expenditure panel survey over 65	Total *N* = 3,658	57.5/44.8% females (exp/contr), mean age 74.8/73.9 yrs for exp/contr	Logistic regression	Socioeconomic status, region, comorbidity index, SF-12 score, BMI, metropolitan statistical area, smoking
Mangtani, 2005	United Kingdom	Cohort study	Dementia and depression (questionnaires)	Influenza (last year)	Participants over 74 from MCR trial of assessment and management of older people in the community	Total *N* = 5,572, Follow-up time 5yrs	47% females, adults > 74 yrs	descriptive	None
Peytremann-Bridenvaux, 2008	Austria, Denmark, France, Germany, Greece, Italy, Netherlands, Spain, Sweden and Switzerland	Cross-sectional	Depression (questionnaire)	Influenza (last year)	Participants over 65 in the survey of Health, Ageing, and Retirement in 10 different European countries	Total *N* = 15,380	70.5/48.3% females (exp/contr) 56.4/46% over 65 yrs (exp/contr)	Logistic regression	Age, gender, socioeconomic status, behavioural risk, chronic disease, disability, country of residence
Shah, 2012	United Kingdom	Cross-sectional	Dementia (Read codes)	Influenza (last year)	Patients registered in The Health Improvement Network (THIN) over 65 years	N (community) = 5,048 N (care homes) = 3,582	87.6/63.2% females (care homes/community-dwelling), mean age 85.5/74.7 yrs for care home/community	Logistic regression	Age, gender, community setting, registration length in care home, deprivation, comorbidities
Thorpe, 2006	United States	Cross-sectional	Psychological distress (questionnaire)	Influenza (last year)	Participants over 65 in the Medical Expenditure Panel Survey	Total N = 3,566	58% females, mean age 74.4 yrs	Logistic regression	Age, gender, ethnicity, marital status, education, risk taking attitudes, beliefs about value of medical care, region, metropolitan area, physical health
Adults									
Nicolaidis, 2012	United States	Cross-sectional	Autism spectrum disorder (self-reported and questionnaires)	Tetanus vaccine within the last 10 years	Online convenience sample	Total *N* = 437	36.7/42.2% females (exp/contr), mean age 37.3/39.7 yrs (SD 12.9/12.9) for exp/contr	Logistic regression	Age, sex, ethnicity, personal and parental education, income, health insurance, overall health status
Xiang, 2015	United States	Cohort study	Depression (questionnaire)	Influenza vaccine within the last two years	Participants over 50, enrolled in primary care register, without chronic disease at start	Total *N* = 11,439	55.4% females, mean age 61.4 yrs	Logistic regression	Age, gender, ethnicity, education, marital status, employment status, household wealth, activity of daily living difficulties, any chronic disease
Mother and their children									
Gilbert, 2021	United States	Cross-sectional	General mental health score (questionnaire)	Childhood immunisation (not further specified)	Low-income mothers, enrolled in other longitudinal study	Total *N* = 843	100% females, mean age 28.8yrs (SD 6.0)	Logistic regression	Mother’s age, child’s age, ethnicity, mother’s education
Lyman, 2008	United States	Cohort study	Depression (questionnaire)	IPV, MMR and DTP vaccine at 24 months	Participants of the national maternal and infant health survey	Total N (mother baby pairs) = 7,296	59.1% females, mean age 70.8 yrs (SD 4.4)	Logistic regression	Poverty, single-parenthood, marital status, maternal ethnicity, wantedness of child, education, employment, health insurance of the child
Lyngsoe, 2018	Denmark	Cohort study	Depression (diagnoses and medication in health records)	DTP and MMR at 24 months	Mothers and their children in the Danish primary care data base	N (Children) = 850,243 N (Mothers) = 517,107 Follow-up time 13yrs	100% females, mean age 30.6 yrs (SD 4.9)	Poisson regression	Household income, parents occupation and education level, number of siblings, maternal age at birth, mental health comorbidity, paternal depression
Minkovitz, 2004	United States	Cohort Study	Depression (questionnaire)	DTP, IPV, MMR at 24 months	Family samples from the Healthy steps for young children cohort study	Total N (families) = 5,565 Follow-up time 3 yrs	100% females, 13.3% < 20 yrs, 50.5% 20–29 yrs, 36.2% > 30 yrs	Logistic regression	Mother’s age and ethnicity, parent’s employment status, child’s gender, household income, home ownership, children’s insurance status, birth weight, child’s health status, enrolment site
Osam, 2020	United Kingdom	Cohort study	Depression, anxiety, psychosis, eating disorder, personality disorder, alcohol and drug use (read codes)	MMR and 5-in-1 booster at age 2 and 5	Mothers and their children in nationwide primary care data set (CPRD)	N (mothers) after 2 years = 479,949 N (mothers) after 5 years = 326,082 Follow-up time 12 yrs	100% females, mean age 30.3 yrs (SD 5.8)	Logistic regression	Sex of child, child’s ethnicity, delivery year, maternal age, socioeconomic status, region
Turner, 2003	Australia	Cohort study	Depression, anxiety or combination	DTP, IPV, HiB at 7 months	Women from the Royal Women’s Hospital in Brisbane	Total N (mothers) = 159 Follow-up time = 6 months	100% females, mean age 30.3 yrs (range 17–42 yrs)	Logistic regression	Mother’s age and income
Children									
Ankustiri, 2012	United States	Cohort study	Autism spectrum disorder	HBV, DTP. PCV, IPV, MMR completed up to age 5	Children participating in the Autism Phenome Project	Total N = 240 Follow-up time 5 yrs	18% females, age between 2–5 yrs	Descriptive analysis	None
Kuwaik, 2006	Canada	Cross-sectional	Autism spectrum disorder (DSM-IV)	MMR at 12 months, DTP \t 2, 4, 6, and 18 months	Sub-sample from Canadian Autism Cohort study	N (children) = 98 N (siblings) = 98	32.6% females, age between 0-18yrs	ANOVA for group differences	None
Tuffrey, 2001	United Kingdom	Cross-sectional	Learning disability (approximated by special school attendance)	DTP, IPV, MMR, VAR (year 4) HPV, MCV4, DTP (year 11)	Children attending special schools in Bath clinical area	Total *N* = 408	36.7% females, age between 4–16 yrs	Chi-square tests	Maternal age, ethnicity, month and year of birth, child’s sex and birth site (for matching)
Zerbo, 2012	United States	Cohort study	Autism spectrum disorder (ICD-9 codes)	DTP, PCV, IPV, MMR, Var, HPV, MCV4, overall childhood immunisations completed in period of age 1–2 years, 4–6 years and 11–12 years	Members of the integrated health care delivery system	Total *N* = 596,633 Follow-up time 15 yrs	18.1/46% females (exp/contr), 76.5% 4–6 yrs old, 23.5% 11–12 yrs old	Logistic regression	Maternal age, ethnicity, month and year of birth, sex of the child and birth site

For some studies in the elderly population, the total study population may differ from sample considered for vaccine uptake (based on vaccine eligibility) and also may differ between different vaccines as outcome

*MMR* measles mumps and rubella vaccine, *DTP* diphtheria, tetanus and pertussis vaccine, *PCV* pneumococcal vaccine, *IPV* inactivated polio vaccine, *Var* varicella vaccine, *HPV* human papillomavirus vaccine, *MCV4* meningococcal conjugate vaccines, *HiB* haemophilus influenza type b vaccine, *HBV* Hepatitis B vaccine. 5-in-1: combined vaccine for diphtheria, tetanus, pertussis, polio and haemophilus influenza type B.

### Risk of bias assessment

According to Table 2, most studies showed a high risk of selection bias. This was due to selection of subgroups of populations who were already actively engaging with the health care system or covered by a certain health care insurance in the United States.

**Table 2:** Risk of bias assessment

Author	Country	Study design	Risk of confounding	Risk of Selection Bias	Risk of non-differential misclassification of the exposure	Risk of non-differential misclassification of the outcome	Information bias of exposure	Information bias of outcome	Reverse causation
Ankustiri et al. (2012)	United States	cohort study	High	High	NA	NA	NA	Low	NA
Browne et al. (2019)	United States		High	High	Moderate	Low	Moderate	Low	NA
Kuwaik et al. (2014)	Canada		High	High	Low	Moderate	Moderate	Moderate	Low
Lyman (2008)	United States		Low	High	Low	Moderate	Low	High	NA
Lyngsoe et al. (2018)	Denmark		Moderate	Moderate	Moderate	Low	Moderate	Low	Low
Mangtani et al.(2005)	United Kingdom		High	High	Low	Low	Low	High	Moderate
Minkovitz et al. (2004)	United States		Moderate	Moderate	Low	Moderate	High	Low	Low
Osam et al. (2020)	United Kingdom		Low	Moderate	Moderate	Low	Moderate	Low	Low
Turner et al. (2003)	United States		High	Moderate	Low	Low	Low	Low	Low
Xiang (2015)	United States		Moderate	High	Low	High	Low	High	Low
Zerbo et al. (2018)	United States		Moderate	High	Low	Low	Moderate	Low	Low
Bazargan et al. (2020)	United States	cross-sectional study	Low	High	Low	High	Low	High	moderate
Buchwald et al. (2000)	United States		Low	High	NA	Low	Moderate	Low	moderate
Druss et al. (2001)	United States		Low	High	Moderate	Low	Moderate	Low	NA
Gilbert et al. (2021)	United States		Low	High	Moderate	High	Low	High	moderate
Howard et al. (2006)	United States		Low	High	High	High	Moderate	High	moderate
Lawrence et al. (2020)	United States		Low	High	Low	Low	Moderate	Low	moderate
Lin et al. (2016)	United States		Low	High	Low	High	Low	High	moderate
Nicolaidis et al. (2012)	United States		Low	High	High	High	Low	High	Low
Peytremann-Bridenvaux et al. (2008)	10 European countries (Austria, Denmark, France, Germany, Greece, Italy, Netherlands, Spain, Sweden and Switzerland)		Low	High	Low	High	High	High	Moderate
Shah et al. (2012)	United Kingdom		Low	Moderate	Moderate	Low	Moderate	Low	Low
Thorpe et al. (2006)	United States		Low	High	Low	High	Low	High	Moderate
Tuffrey et al. (2001)	United Kingdom		High	High	High	Low	High	Low	Low

The risk of bias rated for each study by different domains of bias. The detailed rating criteria can be found in the supplement (see Table S1)

Potential confounding bias may have resulted from a lack of adjustment in some studies which were not designed for linking mental health with vaccine uptake but were part of surveillance.

Some of the studies from the United States did not provide sufficient information on whether a vaccine was covered by the respective health insurance or looked at Medicare use only which could still require co-payment for some vaccines [25].

A few studies were adjusting for factors closely linked to health care seeking behaviour such as health insurance and health care utilisation which might have resulted in collider bias in those studies. This means that it was adjusted for something which is a consequence of both outcome and exposure, which might result in a distorted association [36]. Whilst most studies on electronic health records provided relatively reliable information on vaccine uptake, those were more likely to suffer from moderate biases towards the diagnosis of health care issues in primary care. The majority of cross-sectional studies assessed all participants for mental health issues using questionnaires but collected data about vaccine receipt by participant self-recall and were consequently potentially biased by differential misclassification of vaccination status.

A graphical bias exploration using a funnel plot (see supplementary Figures S.5, S.6, S.7) indicated some asymmetry. We interpreted this finding as being attributable to the high heterogeneity between the studies caused by very different study populations, different ways of assessing mental health and vaccine receipt.

### Vaccine uptake in different study populations

Overall, a wide variety of different subgroups of the population was covered in the studies. These ranged from elderly adults, general adult population, children and mothers with their children. All effect estimates can be found in the supplement material ordered by the different subgroups (see supplementary Figures S.1, S.2, S.3, S.4).

#### Adults

**Fig. 2:**
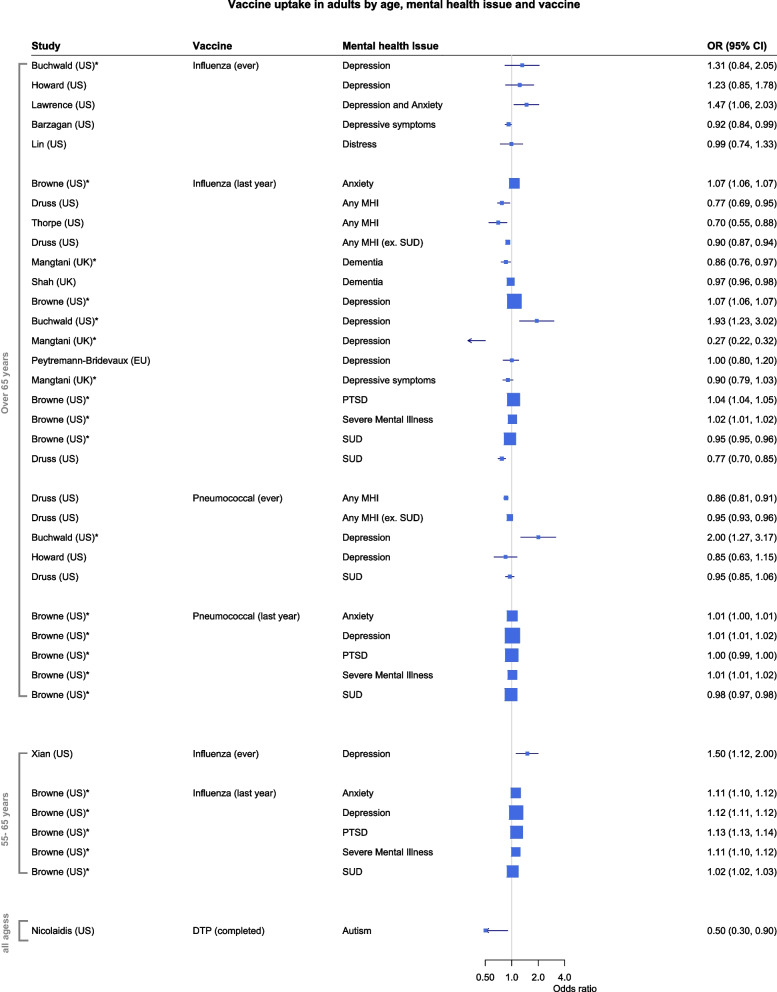
Odds of vaccine uptake in the group with mental health issues in comparison to a control group by age, type of vaccine, and type of mental health issue. MHI: Mental health issue. SUD: Substance use disorder. PTSD: post-traumatic stress disorder. DTP: diphtheria, tetanus, pertussis. *Study which only presented a crude odds ratio without any adjustment for potential confounders

11 studies explored the uptake of influenza and pneumococcal vaccine in populations over 65 years, two studies covered the same vaccines in adults between 50 and 65 years and one explored the uptake of tetanus vaccine in the general population over 18 years. More detailed results broken down by mental health issue and type of vaccine can be found in Fig. 2.

Overall, there were no consistent findings regarding the association between mental health issues and vaccine uptake. In adults over 65, two studies including mental health issues in general or psychological distress found a consistent lower vaccine uptake for all different types of vaccines in comparison to adults with no reported mental health issue [28, 37].

Two other studies showed some evidence for lower Influenza vaccine uptake in community-dwelling individuals with dementia [26, 27]. In addition to this result, Shah et al. [27] differentiated between influenza vaccine uptake in a community setting and in a care home setting, with dementia being a predictor of marginally higher uptake in a care home setting alongside other chronic comorbidities. These were the only two studies for this age group not conducted in the US but in several European countries.

The results for studies in adults between 50 and 65 were consistent with each other, the cohort study in adults with depression controlling for the presence of other chronic diseases [38], and a cross-sectional study in veterans with from anxiety, depression, PTSD, SUD or other severe mental illness showed a higher uptake of Influenza vaccine uptake in comparison to the control group [24].

A single cross-sectional study found a lower uptake of tetanus vaccine within the recommended 10-year time frame for adults with from autism spectrum disorder based on self-rating or external diagnosis [39].

#### Children with mental health issues

Overall, there were four studies looking at mental health issues in children themselves and how they impacted their vaccine uptake. A study in children attending a school for learning abilities in the U.K. showed a non-significant trend for slightly lower uptake of most vaccines and more missed vaccines in comparison to children who did not attend those schools [33].

Three studies looked at children with autism spectrum disorder [34, 35, 40]. There was no difference between the exposed children and the control group for children aged 6 or older. Only [35] found a statistically significant lower uptake in children at the age 5 with autism for all different types of vaccines. For more details see supplementary Figure S1.

#### Siblings of children with autism spectrum disorder

Two studies looked at younger siblings of children with autism in Canada and in the United States [34, 35]. Zerbo et al. [35] found inconclusive results for children older than 6 years. For siblings younger than 6 years, both studies showed consistent results of a higher risk of delaying a vaccine or not being fully vaccinated at different ages. For more details see supplementary Figure S.2.

#### Children of mothers with mental health issues

Six studies covered the impact of maternal mental health on the vaccine uptake in their children [29–32, 41, 42]. For children at the age of five, there were consistent results for a lower odds of receiving a vaccine if they had a mother affected by a mental health issue or with a lower score of mental well-being [29, 31]. This was both in a U.S. and a U.K. setting. However, Gilbert et al. [29] did not find conclusive results for the impact of mental well-being among low-income mothers in delaying their children’s vaccination.

Studies covering the vaccine uptake at the age of two did not show a clear trend of higher or lower uptake. Particularly studies on depression showed opposite results [30, 31, 42]. It should be noted though, that these studies were conducted in different health care setting such as the U.S., Denmark and the U.K.

For younger children under two, a single study from Australia showed a higher risk of delaying vaccines for children of mothers with mental health issues but no higher risk for partially completed vaccination schedules [32]. Minkovitz et al. [42] did not show a statistically significant lower or higher uptake in children at the age one, independent of whether the depression was diagnosed 2–4 months postpartum or later in childhood 30–33 months postpartum. For more details see supplementary Figure S.3.

### Effect of different mental health issues

Expecting different mechanisms of how a mental health issue could impact and influence vaccine uptake in different subgroups, we also explored results grouped by different mental health issues.

Despite the heterogeneity across different studies, several studies found substantial health inequalities for individuals with mental health issues. Osam et al. [31] found a substantially lower vaccine uptake in children of mothers with substance use disorder, similarly adults over 65 with substance use disorder showed a significantly lower influenza uptake in comparison to healthy controls [24, 37]. Some studies found a drastically lower vaccine uptake in people with depression or their children [26, 31].

#### Depression

Depressive disorders and symptoms were the most studied mental health issues across all studies. All studies with depression (or maternal depression, as applicable) as exposure can be found in supplementary Figure S.4.

Definitions of ‘being depressed’ varied between studies from assessing symptoms only, using structured tools, health records, different times of occurrence and depression mixed with anxiety. For maternal depression, studies also differentiated between different onsets of the symptoms. Except two studies of middle-aged adults, there was neither a consistent trend within an age group nor between different definitions for depression nor vaccines.

#### Autism

Siblings of children with autism spectrum disorder showed a lower uptake of different vaccines at younger age as well as higher odds for having an incomplete or delayed vaccination status [34, 35]. This was the case for all different types of vaccines. Two studies found a lower uptake in individuals with autism, Zerbo et al. [35] in children under the age of 6 and Nicolaidis et al. [39] in adults. Both studies were based in a U.S. health care setting.

#### Other mental health issues

More details on the impact of mental health issues can be found in the figures in the supplementary material (Figures S.1, S.2, S.3, S.4).

#### Timing of diagnosing a mental health issue

Two cohort studies explored the effect of time of the occurrence of the mental health issue in relation to the vaccine receipt. Minkovitz et al. [42] found a lower uptake for all types of childhood vaccines if the mother experienced depressive symptoms between 2 and 4 months after birth but not for depressive symptoms between 30 to 33 months after birth. Lyngsøe et al. [30] did not find any difference between mothers having experienced depression in the past and currently experiencing depression. However, most studies did not provide any information about the timing between the occurrence of a mental health issues and when the individuals received their vaccine.

### Different types of vaccine

During childhood, there was no consistent trend in differences of uptake noticeable for different types of vaccines. This also applied to different vaccines for adults of all ages.

### Timeliness of the vaccine

Only four studies explored the effect of mental health issues on vaccine delay or being partially vaccinated [29, 33, 34]. Turner et al. [32] found higher odds of delaying vaccines for children of mothers with mental health issues but not for eventual coverage later. In contrast, Gilbert et al. [29] found that women with lower self-rated mental health status were less likely to have children with up-to-date vaccinations but no difference in delaying vaccination. One study indicated higher odds of being only partially vaccinated for children with learning disability [33] and another study showed both, a lower uptake and a higher proportion of delayed vaccines in younger siblings of children with autism [34].

## Discussion

This is the first review to our knowledge which systematically explored the link between a range of mental health issues and all vaccines recommended across the life course. We identified 23 papers with very different underlying study populations as well as different definitions of the same mental health issue and vaccine receipt.

The results of the studies differed depending on age of the study population, setting, geographical region, and the mental health issue involved. This finding aligns with a previous review by Lord et al. [10], indicating that more context specific research within certain populations is needed. In adults we saw inconsistent results, only for adults between 50 and 65 years there was a consistent trend of having higher odds of getting vaccinated in the group with mental health issues than without. There was no consistent result for children with autism but a trend for missing some vaccines in children with learning abilities. In contrast, younger siblings of children with autism showed consistently lower vaccine uptake across studies. For children of mothers with mental health issues, there was only a trend of being less vaccinated at the age of 5 across different maternal mental health issues. There were no consistent trends across ages for different mental health issues. Nevertheless, some studies found substantial health inequalities in vaccine uptake, e.g. for individuals with substance use disorder or depression [24, 26, 37, 43]. Inconsistent results across studies do not necessarily imply that there is no true health inequality but could be impacted by very different study designs, case definitions, sampling methods, and differences in health care systems.

Overall, the study populations were drawn from very different subpopulations. Our bias assessment indicated that many studies were at risk of selection bias by focusing on a population which already accessed health care services in a certain way. Additionally, many differences can probably be linked to systematic or structural factors that may shape vaccination behaviours such as access to health care, health insurance, or place of residence [44, 45]. This might be especially relevant to studies conducted in the United States where many different types of insurances offer vaccination under varying conditions and sometimes require co-payment [25]. Unfortunately, only some of the U.S. studies presented data on the insurance or Medicare status but did not provide information about whether vaccination were financially covered for individuals. Additionally, personal attitudes and beliefs [46–48], and institutional trust [49] are other factors which might have impacted the individual’s vaccination uptake. Depending on age and other living circumstances, some people might rely more on other people in order to get vaccinated such as children or people with dementia.

A higher vaccine uptake in adults between 50 and 65 years could be due to physical health conditions which made them eligible for influenza and pneumococcal vaccine in the first place [24, 38] and then led to higher ascertainment of mental health issues due to regular health care visits. This theory could be supported by the finding by Shah et al. [27] who only found a higher vaccine uptake in dementia patients living in care homes with more frequent access to health care than community-dwelling dementia patients. The consistent trend of lower vaccine uptake in younger siblings of children with autism [34, 35], [34, 35] could be explained by the parent’s fear that a vaccine might have caused autism in the older sibling following a popular belief after the later disregarded publication by Wakefield [50]. A lower vaccine coverage in children of mothers with mental health issues mainly at the age of 5 might be impacted by barriers to accessing services as there is less structure for the children’s health reviews which usually go along with routine vaccination in the first year of life (e.g., [51]).

Some health care seeking behaviours seem to be influenced by mental health issues, however existing studies in several high-income countries showed inconsistent results, for instance higher health care utilization in patients with anxiety [52] and depression [53], in contrast to lower uptake of other preventive care services in patients with depression [54] or severe mental illness [5]. A U.K. study by Milan & Dáu [55] showed that mothers with traumatic experiences and PTSD had a lower uptake of the COVID-19 vaccine and reported a bigger distrust in institutions than mothers without any mental health issues. Another study by Renbarger et al. [56] illustrated that women with SUD can feel judged and scrutinised by health care professionals and consequently, try to avoid primary care services. Similar experiences could apply to participants of some of the included studies. However, even within groups of mental health issues, heterogeneity in findings remained.

The quality of the included studies varied and many of them were subject of potential selection bias, focusing on populations already actively engaging with the health care system. This might also lead to a misclassification of the exposure as some people might not be diagnosed with their mental health issue due to access or might perceive a diagnosis as stigmatising. Hence, the general impact of mental health on vaccine uptake might be underestimated as more vulnerable groups were not represented or people with mental health issues might be misclassified into the control group.

Overall, this systematic review used a comprehensive search strategy in multiple databases including grey literature, and minimised human error through screening all included studies by two authors and using a comprehensive framework to assess potential bias in all studies. We aimed to address the gap in knowledge of the association of vaccine uptake across a wide range of mental health issues with no constraints to age, subgroup of the population, type of vaccination, or language of the study. Another strength was as an extensive bias assessment of the included studies which identified various patterns of bias across studies which helped to carefully set their results into context.

There are some limitations to our review. Although including studies in all languages, we only conducted our search in English language data bases. We also did not conduct any hand searching of suitable references in the included articles.

Many studies applied different ways of defining a complete vaccination status and documenting vaccine receipt which made the outcome more difficult to compare. Although we included a broad definition of mental health issues and examined all vaccine types in all age groups, there was not enough data from the studies to meta-analyse the results and draw an overall conclusion. Further, defining and diagnosis mental health issues is changing and subject to temporal, cultural and contextual trends [57]. The very different study populations, different health care systems with different access barriers and different ways of defining and ascertaining mental health made any comparison of results across studies extremely difficult. Those differences in health care systems may have also impacted access to health care and vaccination services. This might particularly important as the review was dominated by studies from the US where health insurance has even bigger impact on access to health care in comparison to other high income countries [58]. Furthermore, the temporality between mental health issue and vaccine uptake was often not considered in the existing studies. It remains unclear whether there more recent mental health issues or chronic conditions might be more impactful than a history of mental health issues.

Many included studies had a high risk of selection bias or ascertainment bias. All studies covered only individuals which already accessed the health care services because of their mental health issues, consequently the impact of mental health on vaccination uptake might be underestimated missing those who did not seek help, e.g., due to access barriers or perceived stigma. This is very important as help-seeking behaviour for mental health issues is often related to similar factors impacting vaccine uptake, such as sociodemographic status, gender, ethnicity, age, comorbidities and perceived need of the health care service [59]. This makes the interpretation of the results more complex. Some studies which based the vaccination status on recall only might also suffer from differential misclassification bias. Potential confounding was also additional issue for many studies as they were initially designed for surveillance purposes only. Other studies tended to over-adjust for potential factors on the causal pathway – including access to health care system or other indicators for health care utilisation.

Overall, we found inconsistent findings in this review which indicates that the receipt of vaccination is likely to be shaped by multiple factors including access to health care, vaccine hesitancy, the nature of the mental health issue, the contact with the health care providers amongst others. Generally, there is not much existing evidence on a potential link between mental health issues and vaccination uptake, for both individuals and mothers and their children. However, some studies indicated potentially big health inequalities for individuals with substance use disorder or depression. The burden of mental health issues has remarkably increased in the last decades [4] together with vaccine hesitancy in high-income countries to non-negligible proportions [44] which makes it important to understand whether there is a link between mental health and vaccine hesitancy and/or issues accessing vaccination services in order to prevent lower vaccine-coverage.

There is more research needed on different patterns of vaccine uptake, a better differentiation between mental health issues, their timing in relation to the vaccination, and potential disorder specific issues. In addition, there is a need for clinically evaluated mental health outcomes as the reliability of self-reported diagnoses and symptoms may not accurately classify people. Some groups of people with mental health issues seem to be especially neglected in research despite their higher risk of health issues, for instance people with severe mental illness [5]. The temporality of mental health issues in relation to vaccine uptake was often not considered in existing studies but might be useful to identify individuals at higher risk for missing vaccination. Additionally, a better understanding of mechanisms through which mental health issues might affect vaccine uptake is needed. Our review should motivate future research to investigate how mental health issues may impact individuals access primary health care and vaccination services and to shift research on vaccine uptake in people with mental health issues towards more context and disorder sensitive designs. This can help to better asses potential health inequalities and develop more targeted public health interventions if needed.

## Supplementary Information

**Additional file 1.** 

**Additional file 2: Table S. 1 **Risk of biasassessment tool 

**Additional file 3: Figure S.1 **Vaccine uptake in children with mental healthissues.** Figure S.2 **Vaccine uptake in siblingsof children with autism spectrum disorder.** Figure S.3 V**accine uptake in children of mothers withmental health issues.** Figure S.4  **Vaccine uptake in individuals with depression. **Figure S.5  **Funnel plot for allincluded studies.** Figure S.6 **Funnel plot for all studies covering adults.** FigureS.7 **Funnel plot for all individuals with depression

## Data Availability

Data availability is not applicable to this article as no new data were created or analysed in this study. The extraction table can be requested from the corresponding author (AS). The automated deduplication code can be accessed via GitHub (see link in the methods).
