# Supplementary material for: Exploring the impact of mental health conditions on vaccine uptake in high-income countries: a systematic review
Source: BMC Psychiatry. 2023 Jan 7;23:15. doi: 10.1186/s12888-022-04512-y (PMC9823258; doi:10.1186/s12888-022-04512-y)
Supplement: Supplementary file 1 — Additional file 1. [file 12888_2022_4512_MOESM1_ESM.docx]

## Supplementatry information

## PROSPERO protocol


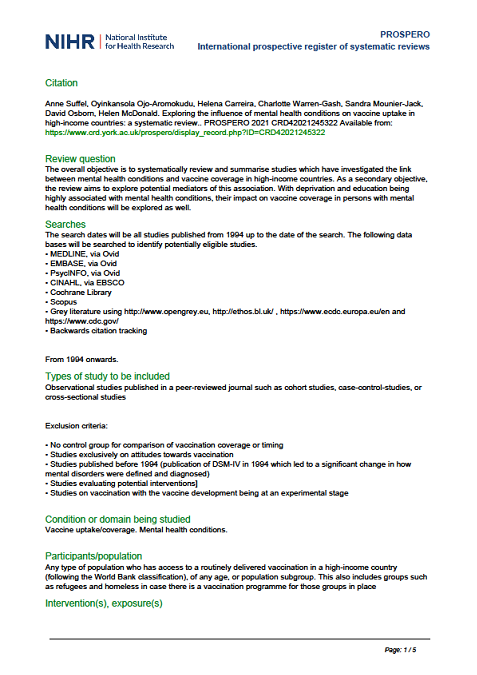


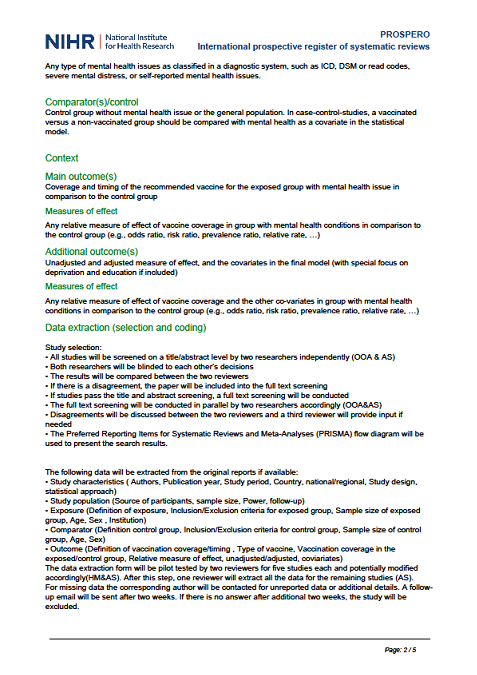


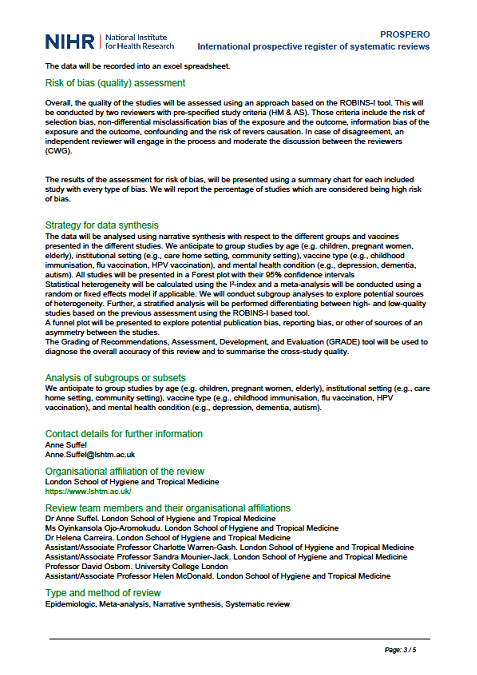


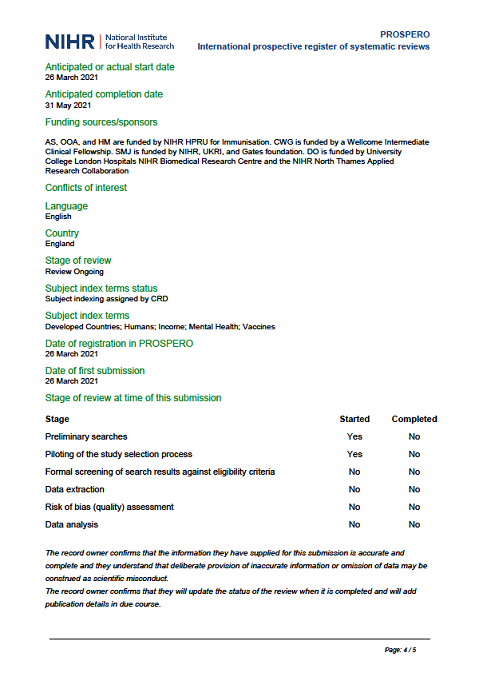


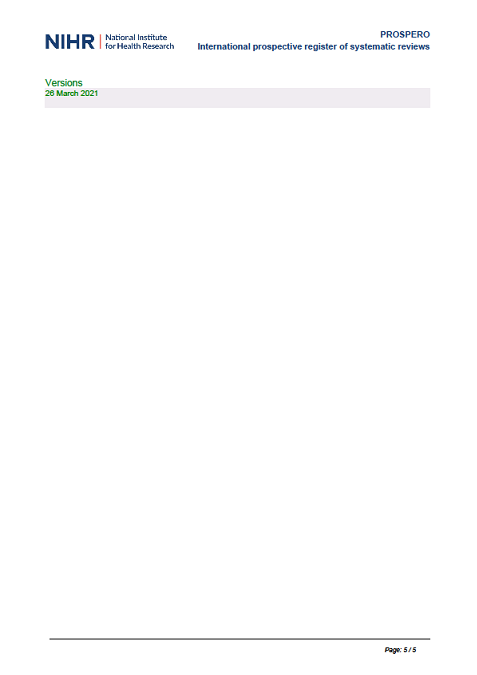


## Search Terms

**Medline (OVID)**

1. ((mental or psychiatric) adj3 (disorder*or illness or health or disease*)).mp.
2. ((substance or alcohol or drug) adj3 (misuse or abuse or dependence)).
3. (psychiatr* or depressi* or mood disorder or anxiet* or phobia* or addiction or psychosis or psychotic or panic disorder* or dementia or schizophren* or schizoaffective or eating disorder* or bulimia or anorexia or binge or post traumatic or PTSD or bipolar or borderline or personality disorder* or fatigue or autism or intellect* disabilit* or sexual dysfunct* or sleep disorder* or attention-deficit hyperactivity disorder* or distress).mp.
4. exp Mental Disorders/
5. 1 or 2 or 3 or 4
6. ((vaccin* or immuni*) adj6 (uptake or accept* or receipt or receive* or preventive* or coverage or rate* or pattern* or seeking)).mp.
7. (preventive medical care or childcare visit* or child care visit*).mp.
8. Vaccination Coverage/
9. 6 or 7 or 8
10. 5 and 9
11. limit 10 ”o yr="1994 -C”rrent"

**Embase (OVID)**

1. ((mental or psychiatric) adj3 (disorder*or illness or health or disease*)).mp.
2. ((substance or alcohol or drug) adj3 (misuse or abuse or dependence))
3. .psychiatr* or depressi* or mood disorder or anxiet* or phobia* or addiction or psychosis or psychotic or panic disorder* or dementia or schizophren* or schizoaffective or eating disorder* or bulimia or anorexia or binge or post traumatic or PTSD or bipolar or borderline or personality disorder* or fatigue or autism or intellect * disabilit* or sexual dysfunct* or sleep disorder* or attention-deficit hyperactivity disorder* or distress).mp.
4. exp mental disease/
5. 1 or 2 or 3 or 4
6. ((vaccin* or immuni*) adj6 (uptake or accept* or receipt or receive* or preventive* or coverage or rate* or pattern* or seeking)).mp.
7. (preventive medical care or childcare visit* or child care visit*).mp.
8. Vaccination Coverage/
9. 6 or 7 or 8
10. 5 and 9
11. imit 10 ”o yr="1994 -C”rrent"

**PsychINFO (OVID)**

1. ((mental or psychiatric) adj3 (disorder*or illness or health or disease*)).mp.
2. ((substance or alcohol or drug) adj3 (misuse or abuse or dependence))
3. psychiatr* or depressi* or mood disorder or anxiet* or phobia* or addiction or psychosis or psychotic or panic disorder* or dementia or schizophren* or schizoaffective or eating disorder* or bulimia or anorexia or binge or post traumatic or PTSD or bipolar or borderline or personality disorder* or fatigue or autism or intellemmuneityabilit* or sexual dysfunct* or sleep disorder* or attention-deficit hyperactivity disorder* or distress).mp.
4. exp Mental Disorders/
5. 1 or 2 or 3 or 4
6. ((vaccin*or immuni*) adj6 (uptake or accept* or receipt or receive* or preventive* or coverage or rate* or pattern* or seeking)).mp.
7. (preventive medical care or childcare visit* or child care visit*).mp.
8. 6 or 7
9. 5 and 8
10. limit 10 ”o yr="1994 -C”rrent"

**CINHAL (EBSCO)**

1. ((mental or psychiatric) N3 (disorder*or illness or health or disease*))
2. ((substance or alcohol or drug) N3 (misuse or abuse or dependen*)
3. psychiatr* or depressi* or mood disorder or anxiet* or phobia* or addiction or psychosis or psychotic or panic disorder* or dementia or schizophren* or schizoaffective or eating disorder* or bulimia or anorexia or binge or post traumatic or PTSD or bipolar or borderline or personality disorder* or fatigue or autism or intellect* disabilit* or sexual dysfunct* or sleep disorder* or attention-deficit hyperactivity disorder* or distress)“
4. (MH "Behavioral and Mental Diso”ders+")
5. 1 or 2 or 3 or 4
6. ((vaccin* or immuni*) N6 (uptake or accept* or receipt or receive* or preventive* or coverage or rate* or pattern* or seeking))
7. (preventive medical care or childcare visit* or child care visit*)“
8. (MH "Vaccination Co”erage")
9. 6 or 7 or 8
10. 5 and 9
11. limit 10 ”o yr="1994 -C”rrent"

**Scopus**

1. ((mental or psychiatric) W/3 (disorder*or illness or health or disease*))
2. ((substance or alcohol or drug) W/3 (misuse or abuse or dependenc*)
3. psychiatr* or depressi* or mood disorder or anxiet* or phobia* or addiction or psychosis or psychotic or panic disorder* or dementia or schizophren* or schizoaffective or eating disorder* or bulimia or anorexia or binge or post traumatic or PTSD or bipolar or borderline or personality disorder* or fatigue or autism or intellect * disabilit* or sexual dysfunct* or sleep disorder* or attention-deficit hyperactivity disorder* or distress)
4. 1 or 2 or 3 or 4
5. ((vaccin* or immuni*) W/6 (uptake or accept* or receipt or receive* or preventive* or coverage or rate* or pattern* or seeking))
6. (preventive medical care or childcare visit* or child care visit*)
7. 6 or 7 or 8
8. 5 and 9
9. limit 10 ”o yr="1994 -C”rrent"

**Cochrane Library**

1. ((mental or psychiatric) NEAR/3 (disorder*or illness or health or disease*))
2. ((substance or alcohol or drug) NEAR/3 (misuse or abuse or dependen*)
3. psychiatr* or depressi* or mood disorder or anxiet* or phobia* or addiction or psychosis or psychotic or panic disorder* or dementia or schizophren* or schizoaffective or eating disorder* or bulimia or anorexia or binge or post traumatic or PTSD or bipolar or borderline or personality disorder* or fatigue or autism or intellect *disabilit* or sexual dysfunct* or sleep disorder* or attention-deficit hyperactivity disorder* or distress)
4. MeSH descriptor: [Mental Disorders] explode all trees
5. #1 OR #2 OR #3 OR #4
6. ((vaccin* or immuni*) NEAR/6 (uptake or accept* or receipt or receive* or preventive* or coverage or rate* or pattern* or seeking))
7. (preventive medical care or childcare visit* or child care visit*)
8. MeSH descriptor: [Vaccination Coverage] explode all trees
9. #6 OR #7 OR #8
10. 5 and 9
11. limit 10 ”o yr="1994 -C”rrent"

**OpenGrey**

((mental or psychiatric) NEAR/3 (disorder*or illness or health or disease*)) OR ((substance or alcohol or drug) NEAR/3 (misuse or abuse or dependence) or psychiatr* or depressi* or mood disorder or anxiet* or phobia* or addiction or psychosis or psychotic or panic disorder* or dementia or schizophren* or schizoaffective or eating disorder* or bulimia or anorexia or binge or post traumatic or PTSD or bipolar or borderline or personality disorder* or fatigue or autism or intellect* disabilit* or sexual dysfunct* or sleep disorder* or attention-deficit hyperactivity disorder* or distress)) AND ((vaccin* or immuni*) NEAR/6 (uptake or accept* or receipt or receive* or preventive* or coverage or rate* or pattern* or seeking))  OR (preventive medical care or childcare visit* or child care visit*))

## Extracted items

- Study characteristics
  - Authors
  - Publication year
  - Study period (years)
  - Country, national/regional
  - Study design
  - Main statistical approach
  - Study population
  - Source of participants
  - Overall sample size
  - Power of the study (if calculated)
  - Time of follow-up
- Exposure:
  - Definition of exposure
  - Inclusion criteria for exposed group
  - Exclusion criteria for exposed group
  - Sample size of exposed group
  - Age (mean/median or min/max)
  - Sex (proportion females)
  - Institution
- Comparator:
  - Definition control group
  - Inclusion criteria for control group
  - Exclusion criteria for control group
  - Sample size of control group
  - Age (mean/median)
  - Sex (proportion female)
- Outcome:
  - Definition of vaccination coverage or timing of vaccination
  - Type of vaccine
  - Vaccination coverage in the exposed group
  - Vaccination coverage in the control group
  - Relative measure of effect, unadjusted
  - 95% Confidence interval of unadjusted measure of effect
  - Relative measure of effect, adjusted
  - 95% Confidence interval of adjusted measure of effect
  - Covariates included in final model
  - Measure of effect for included covariates (including 95% CI)
