# Supplementary material for: Exploring the impact of mental health conditions on vaccine uptake in high-income countries: a systematic review
Source: BMC Psychiatry. 2023 Jan 7;23:15. doi: 10.1186/s12888-022-04512-y (PMC9823258; doi:10.1186/s12888-022-04512-y)
Supplement: Supplementary file 2 — Additional file 2: Table S. 1 Risk of biasassessment tool [file 12888_2022_4512_MOESM2_ESM.docx]

## S. 1 Risk of bias assessment tool

|  | **Low risk** | **Moderate risk** | **High risk** | **N/A** |
| --- | --- | --- | --- | --- |
| **Selection Bias** | | | | |
| **Study population (cohort)** | Study population drawn from population eligible for vaccination and not selected on the basis of access to or use of mental health or preventive care services  Any sampling technique is accounted for in analysis | Study population recruited from a bigger cohort study/Biobank with a higher health surveillance during the study period than the general population  Recruited in a way that reflects their attitudes towards health care or followed up which influenced their health-seeking behaviour | Controls are collected from a different health care setting, or recruitment of cases and controls differs in a way that is likely to be associated with mental health status  Study population recruited from a preventive care centre |  |
| Selection of Controls (case-control) | Controls drawn from population eligible for vaccination and not selected on the basis of use of mental health or preventive care services  Any sampling technique is accounted for in analysis | Recruited in a way that reflects their attitudes towards health care | Controls are collected from a different health care setting, or recruitment of cases and controls differs in a way that is likely to be associated with mental health status  Study population recruited from a preventive care centre |  |
| Participation bias | Participation not depending on active engagement with health care service  Over 80% participation or 70% participation with no systematic difference between those included and not included in the study | Automatic participation using health records  Paid participation | Participants recruited at point of vaccination | N/A in cohort studies |
| Loss to follow up | Automated by linking health records  >80% follow-up  Or no less than 70% follow-up with no systematic difference between those included and excluded in the study (sensitivity check) | 60-79% follow-up | Less than 60% follow up  Systematic differences in those lost to follow up and those still included in the study | N/A in case control studies |
| **Non-differential misclassification of exposure** | Diagnostic interviews by professional to classify mental health status  Validated tool (including from medical record or self-report e.g. depression scale) | Mental health issues defined using health records | Mental health issued defined using recorded symptoms, no validation/sensitivity or other check of mental health definition  Self-report without clear definition? |  |
| **Information bias of the exposure** | | | | |
| Recall bias | Prospectively collected health care data on mental health issues |  | Mental health status defined by patients retrospectively |  |
| Observer bias | Structured interview or diagnosis tools used for mental health diagnosis |  | Incentivised diagnosing of mental health issues |  |
| Ascertainment | All participants were screened for a mental health issue | Mental health issues defined based on electronic health records | Access to mental health services is not covered in the health care system  (Study based in private mental health institutions or services) |  |
| **Non-differential misclassification of the outcome** | Observation of the vaccine administration or active participation of practises  Clear definition of what is ‘vaccinated’ and ‘not vaccinated’  Electronic records of vaccine administration |  | Recall of vaccination by either patient or vaccinator |  |
| **Information bias of the outcome** | | | | |
| Recall bias | Vaccination documented using electronic health records at day of vaccination | Vaccination status recalled by patient | Vaccination status recalled by patient under circumstances which are likely to be affected by mental health status |  |
| Observer bias | Vaccination status documented by default (e.g., electronic health record) |  | Vaccination status documented by health care professionals who is not blinded to the mental health status |  |
| Ascertainment | Objective outcome, clearly defined |  | Less objective outcome, e.g. vaccine given late, and researcher not blinded to exposure |  |
| **Confounding** | | | | |
| **Confounding** | At least age, sex, and ethnicity should be eligible and considered for confounding in the final model  Appropriate statistical model for confounding, discussion of residual confounding  Trends in time considered if relevant (for example changing incentives of diagnosing MHI, Vaccination ‘scandals’) | At least age, sex, and ethnicity should be eligible and considered for confounding in the final model  Appropriate statistical model for confounding, discussion of residual confounding | It was not controlled for more than one of the suggested confounders |  |
| **Reversed causality** | | | | |
| **vaccination status preceded mental health diagnosis (affecting potential causal relationship)** | cohort study  Clear that mental health diagnosis preceded vaccination | No information on timing of mental health diagnosis  Cross-sectional studies: assessing mental health diagnosis and vaccination at the same time | Mental health condition diagnosed after event of vaccination |  |
