# Supplementary material for: Exploring the impact of mental health conditions on vaccine uptake in high-income countries: a systematic review
Source: BMC Psychiatry. 2023 Jan 7;23:15. doi: 10.1186/s12888-022-04512-y (PMC9823258; doi:10.1186/s12888-022-04512-y)
Supplement: Supplementary file 3 — Additional file 3: Figure S.1 Vaccine uptake in children with mental healthissues. Figure S.2 Vaccine uptake in siblingsof children with autism spectrum disorder. Figure S.3 Vaccine uptake in children of mothers withmental health issues. Figure S.4 Vaccine uptake in individuals with depression. Figure S.5 Funnel plot for allincluded studies. Figure S.6 Funnel plot for all studies covering adults. FigureS.7 Funnel plot for all individuals with depression [file 12888_2022_4512_MOESM3_ESM.docx]

## Figure S.1 - Vaccine uptake in children with mental health issues


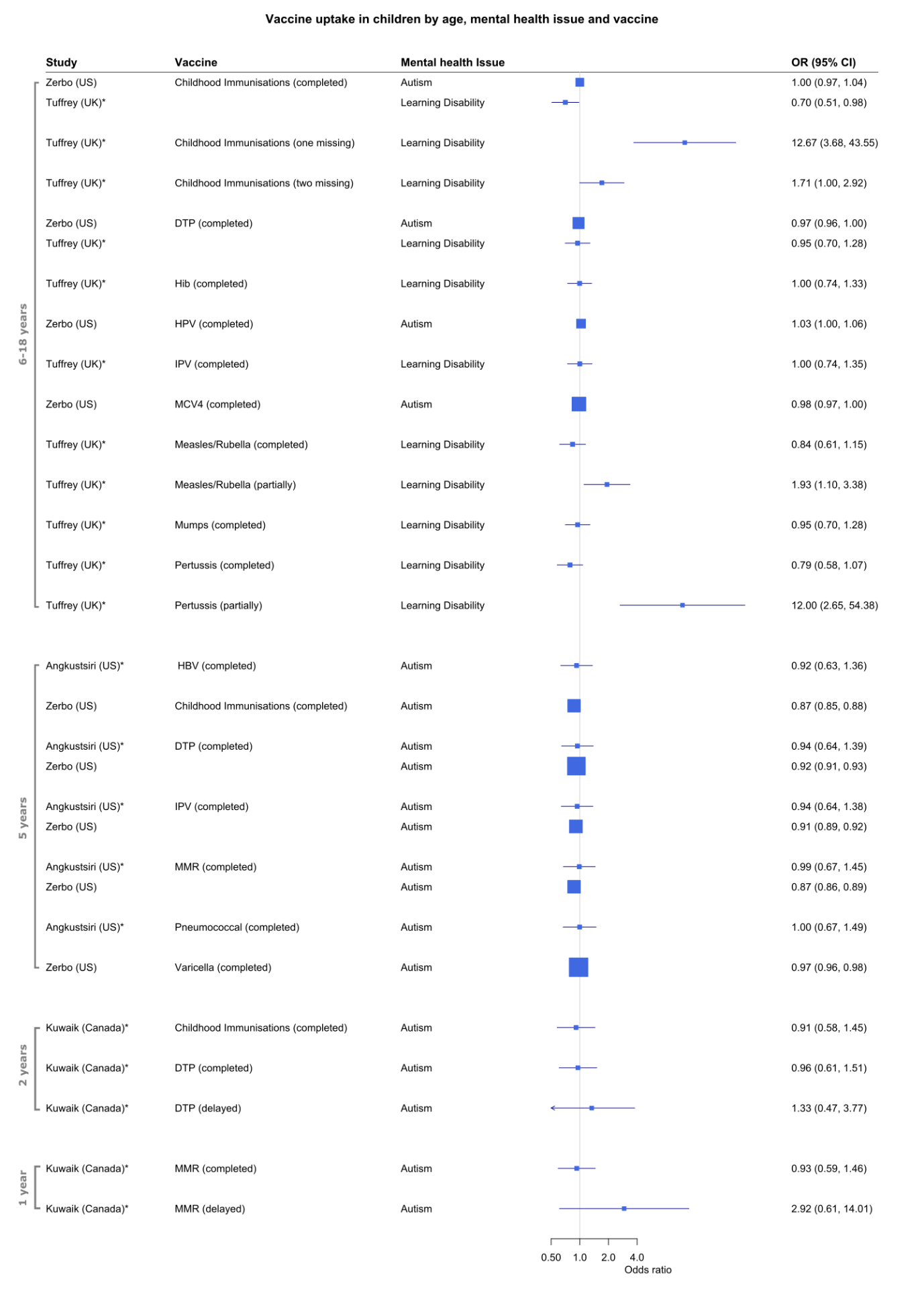


**Figure S.1: Vaccine uptake in children with mental health issues.** MMR: measles mumps and rubella vaccine. DTP: diphtheria, tetanus and pertussis vaccine. PCV: pneumococcal vaccine. IPV: inactivated polio vaccine. Var: varicella vaccine. HPV: human papillomavirus vaccine. MCV4: meningococcal conjugate vaccines. HiB: haemophilus influenza type b vaccine. HBV: Hepatitis B vaccine. 5-in-1: combined vaccine for diphtheria, tetanus, pertussis, polio and haemophilus influenza type B

## Figure S.2 - Vaccine uptake in siblings of children with autism spectrum disorder


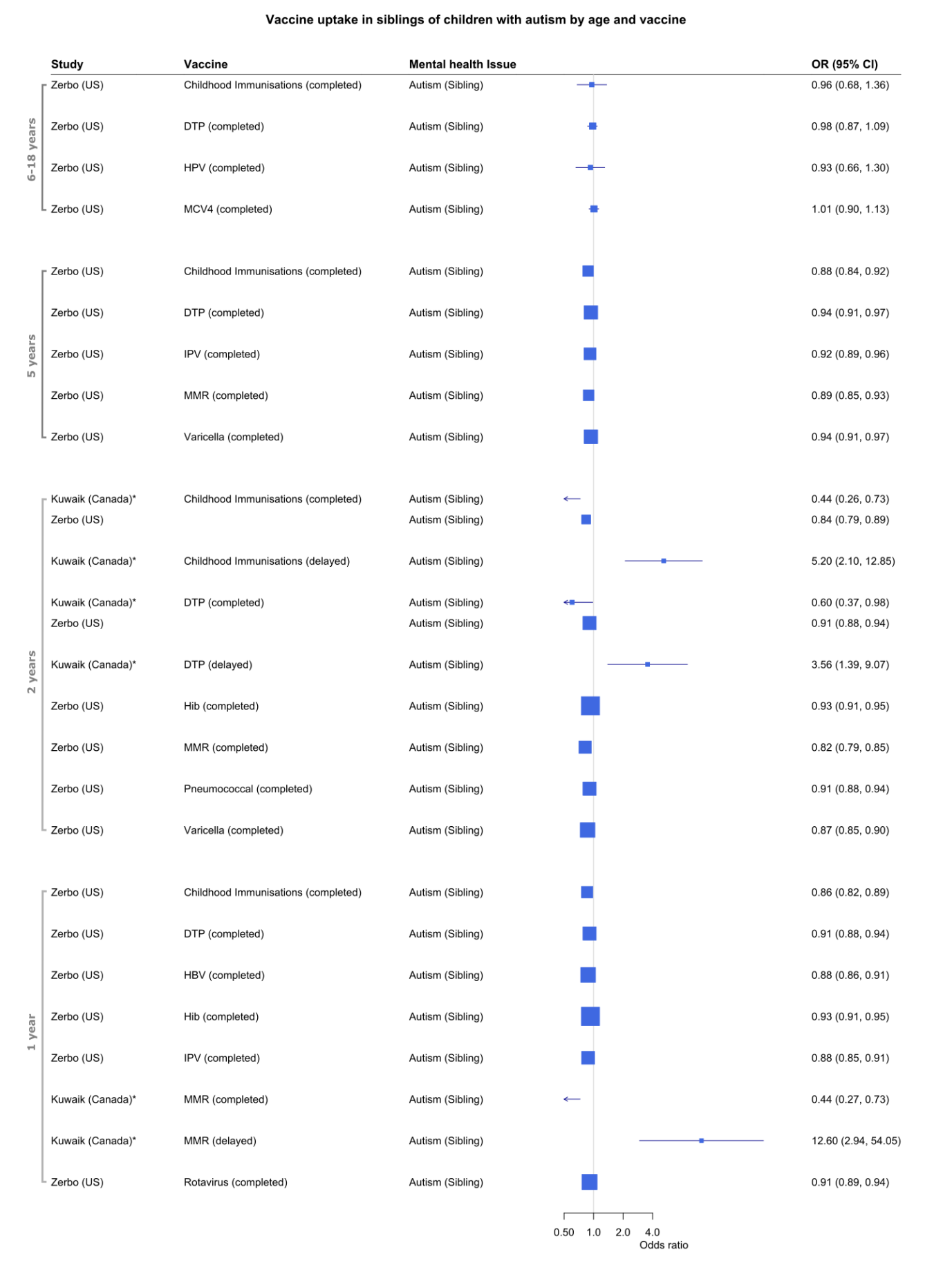


**Figure S.2 : Vaccine uptake in siblings of children with autism spectrum disorder.** MMR: measles mumps and rubella vaccine. DTP: diphtheria, tetanus and pertussis vaccine. PCV: pneumococcal vaccine. IPV: inactivated polio vaccine. Var: varicella vaccine. HPV: human papillomavirus vaccine. MCV4: meningococcal conjugate vaccines. HiB: haemophilus influenza type b vaccine. HBV: Hepatitis B vaccine. 5-in-1: combined vaccine for diphtheria, tetanus, pertussis, polio and haemophilus influenza type B

## Figure S.3 - Vaccine uptake in children of mothers with mental health issues


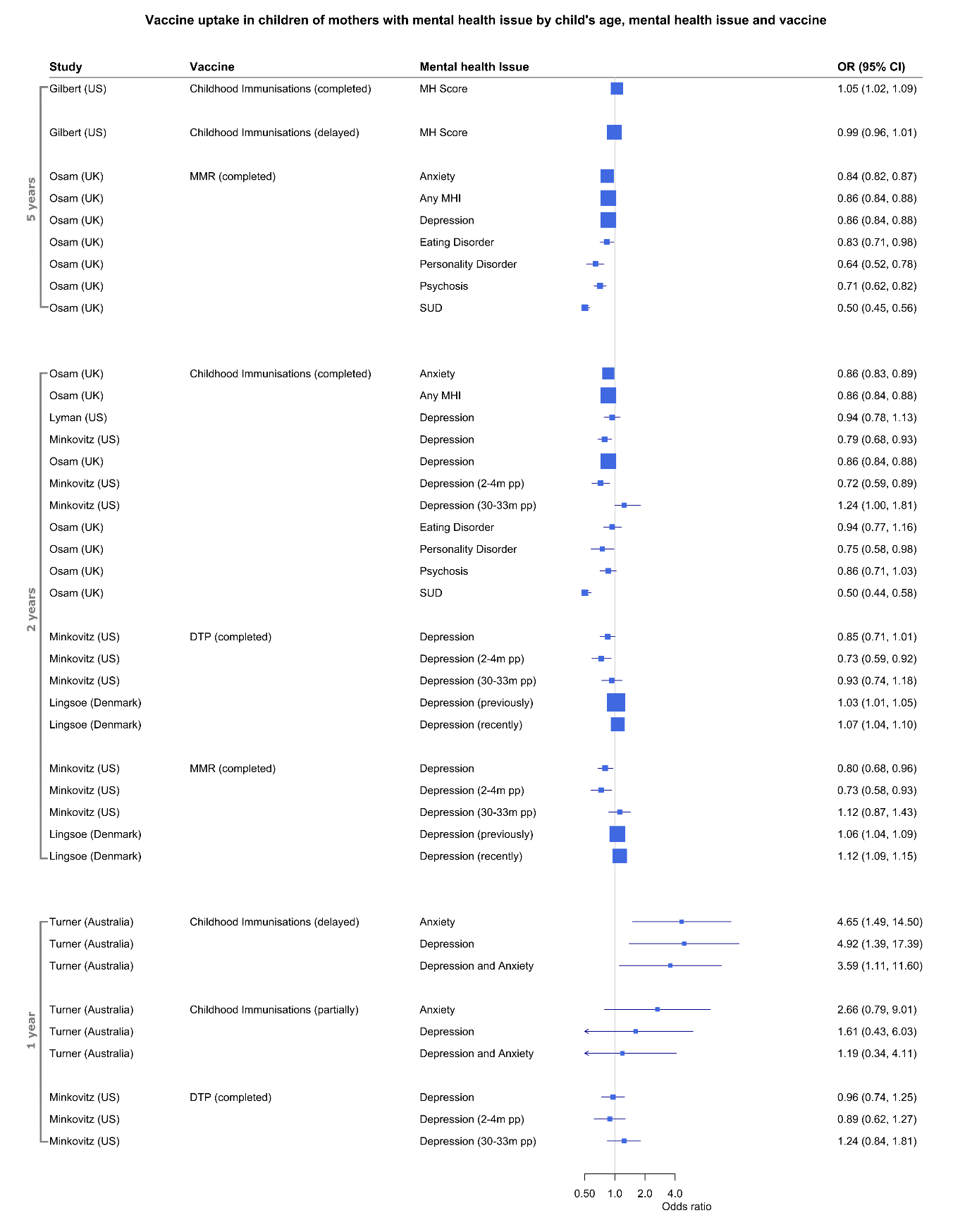


**Figure S.3: Vaccine uptake in children of mothers with mental health issues.** MMR: measles mumps and rubella vaccine. DTP: diphtheria, tetanus and pertussis vaccine. PCV: pneumococcal vaccine. IPV: inactivated polio vaccine. Var: varicella vaccine. HPV: human papillomavirus vaccine. MCV4: meningococcal conjugate vaccines. HiB: haemophilus influenza type b vaccine. HBV: Hepatitis B vaccine. 5-in-1: combined vaccine for diphtheria, tetanus, pertussis, polio and haemophilus influenza type B.

## Figure S.4 - Vaccine uptake in individuals with depression


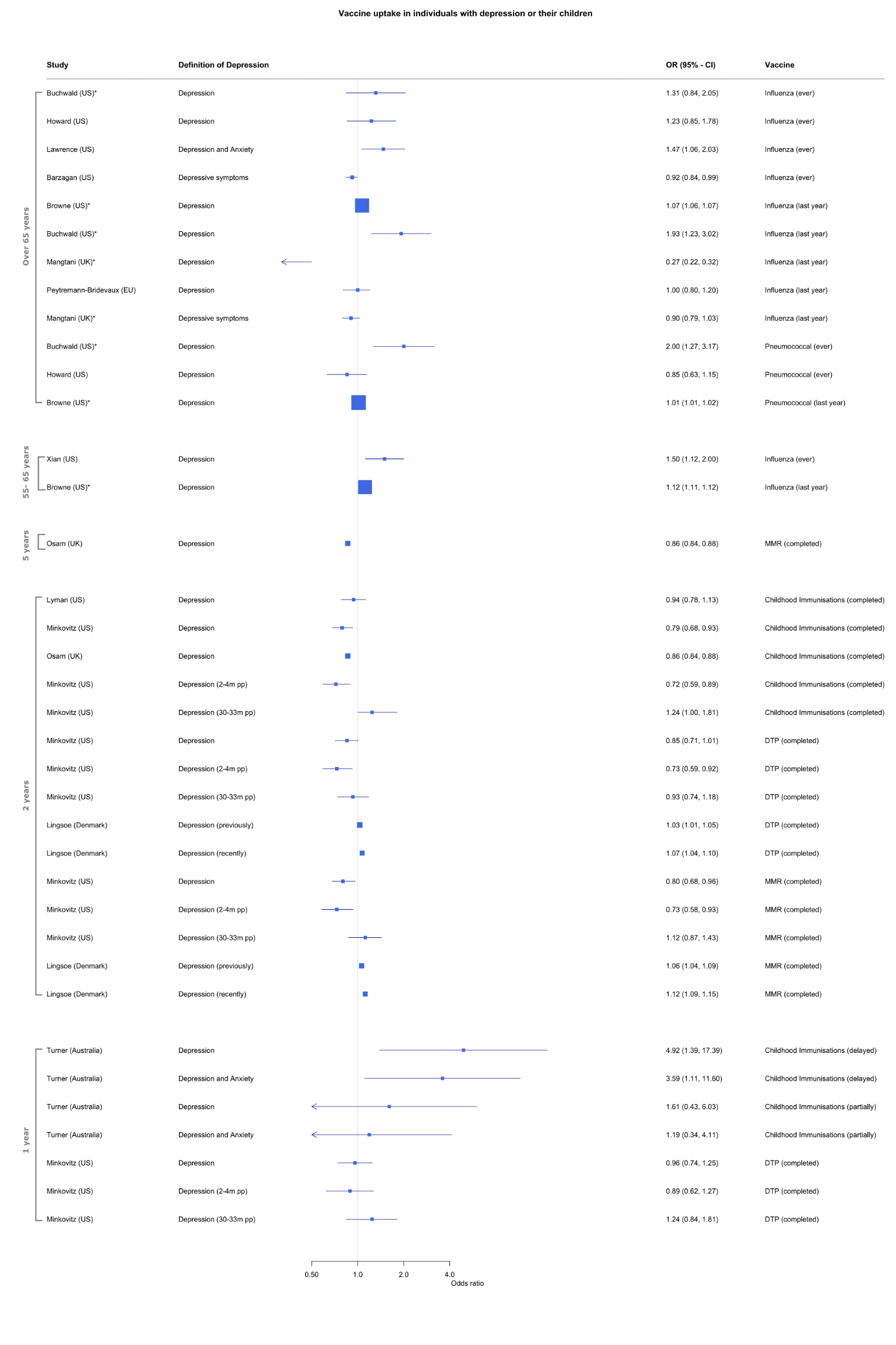


**Figure S.4: Depression and vaccine uptake**. MMR: measles mumps and rubella vaccine. DTP: diphtheria, tetanus and pertussis vaccine. PCV: pneumococcal vaccine. IPV: inactivated polio vaccine. Var: varicella vaccine. HPV: human papillomavirus vaccine. MCV4: meningococcal conjugate vaccines. HiB: haemophilus influenza type b vaccine. HBV: Hepatitis B vaccine. 5-in-1: combined vaccine for diphtheria, tetanus, pertussis, polio and haemophilus influenza type B

## Figure S.5 – Funnel plot for all included studies.

Funnel plot for all included studies:


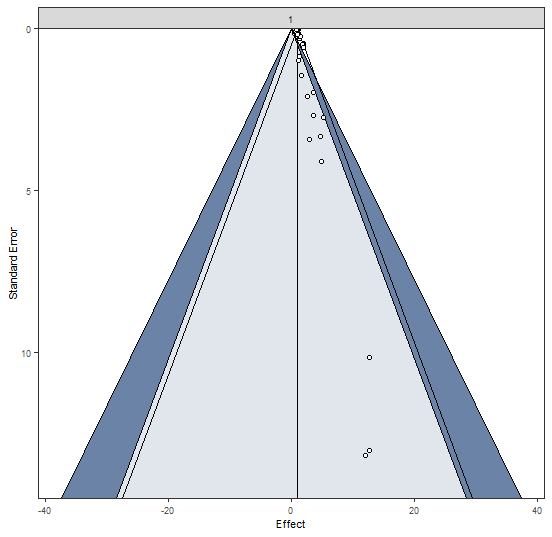


**Figure S.5**: Funnel plot for all included studies.

## Figure S.6 – Funnel plot for all studies covering adults


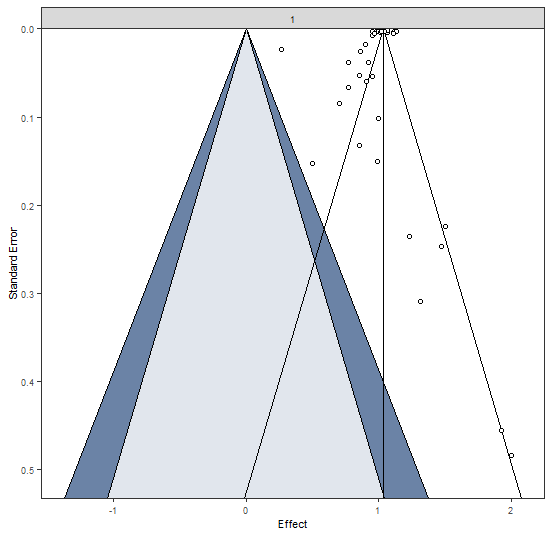


**Figure S.6**: Funnel plot for all studies covering adults.

## Figure S.7 – Funnel plot for all individuals with depression


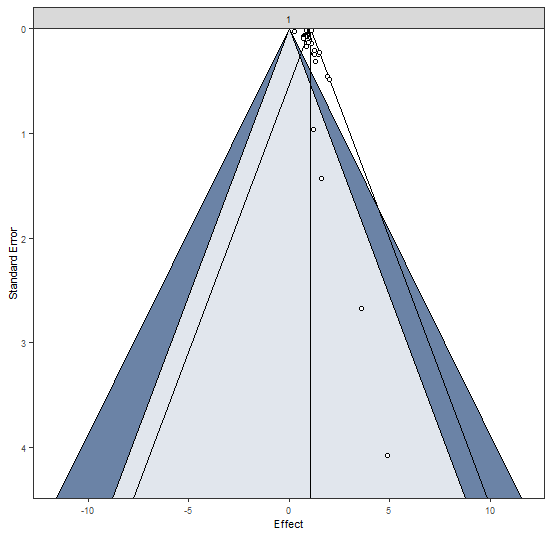


**Figure S.7**: Funnel plot for all studies covering individuals with depression.
